# Supplementary material for: Noise modulation in retinoic acid signaling sharpens segmental boundaries of gene expression in the embryonic zebrafish hindbrain
Source: eLife. 2016 Apr 12;5:e14034. doi: 10.7554/eLife.14034 (PMC4829421; doi:10.7554/eLife.14034)
Supplement: Supplementary file 1. — (B) Modeling parameters DOI: http://dx.doi.org/10.7554/eLife.14034.014 [file elife-14034-supp1.docx]

**Supplementary File 1A - Stochastic Mathematical Modeling**

The stochastic model is similar to the stochastic models previously used for both RA and its target gene expression (White et al., 2007; Cai et al., 2012; Zhang et al., 2012). Below is the basic stochastic PDE model:

$$\frac{\partial\left[ RA \right]_{out}}{\partial t}=D\Delta\left[ RA \right]_{out}+V\left( x \right)-\left( 1+\beta\right)k_{p}\left[ RA \right]_{out}+k_{p}\left[ RA \right]_{in}+\varepsilon_{out}\left[ RA \right]_{out}\frac{\partial^{3}W_{out}}{\partial t\partial x\partial y}$$

$$\frac{\partial\left[ RA \right]_{in}}{\partial t}=k_{p}\left[ RA \right]_{out}-k_{p}\left[ RA \right]_{in}+r_{deg2}\left[ RA-R \right]+bp_{deg2}\left[ RA-BP \right]-{[CYP]\left[ RA \right]}_{in}-r_{on}\left[ RA \right]_{in}\left[ R \right]+r_{off}\left[ RA-R \right]-m_{on}\left[ RA \right]_{in}\left[ BP \right]+m_{off}\left[ RA-BP \right]+\varepsilon_{in}\left[ RA \right]_{in}\frac{\partial^{3}W_{in}}{\partial t\partial x\partial y}$$

$$\frac{\partial[R]}{\partial t}=V_{R}-r_{deg1}\left[ R \right]-r_{on}\left[ RA \right]_{in}\left[ R \right]+r_{off}\left[ RA-R \right]-j_{\alpha}\left[ RA-BP \right]\left[ R \right]+j_{\beta}\left[ BP \right]\left[ RA-R \right]$$

$$\frac{\partial[RA-R]}{\partial t}=-r_{deg2}\left[ RA-R \right]+r_{on}\left[ RA \right]_{in}\left[ R \right]-r_{off}\left[ RA-R \right]+j_{\alpha}\left[ RA-BP \right]\left[ R \right]-j_{\beta}\left[ BP \right]\left[ RA-R \right]$$

$$\frac{\partial[BP]}{\partial t}=V_{BP}-{bp}_{deg1}\left[ BP \right]+\left[ CYP \right]\left[ RA-BP \right]-m_{on}\left[ RA \right]_{in}\left[ BP \right]+m_{off}\left[ RA-BP \right]+j_{\alpha}\left[ RA-BP \right]\left[ R \right]-j_{\beta}\left[ BP \right]\left[ RA-R \right]$$

$$\frac{\partial\left[ RA-BP \right]}{\partial t}=-\left[ CYP \right]\left[ RA-BP \right]+m_{on}\left[ RA \right]_{in}\left[ BP \right]-m_{off}\left[ RA-BP \right]-j_{\alpha}\left[ RA-BP \right]\left[ R \right]+j_{\beta}\left[ BP \right]\left[ RA-R \right]-{bp}_{deg2}\left[ RA-BP \right]$$

where $\left[ RA \right]_{out}$and $\left[ RA \right]_{in}$ represent extracellular and intracellular RA concentrations, and $\frac{\partial^{3}W_{out}\left( t,x,y \right)}{\partial t\partial x\partial y}$and $\frac{\partial^{2}W_{in}\left( t,x,y \right)}{\partial t\partial x\partial y}$denote the space-time white noises in extracellular and intracellular RA concentrations with small positive parameters $\varepsilon_{out}$ and $\varepsilon_{in}$, respectively. $\left[ BP \right], \left[ R \right], \left[ RA-R \right]$and $\left[ RA-BP \right]$ denote the concentrations of binding proteins (Crabps), RA receptors, the complex of RA and receptors, and the complex of RA and binding proteins.

$\left[ Cyp \right]$represents RA degradation by Cyp26 through the following form,

$$\left[ Cyp \right]=\left\{ \begin{matrix} k_{deg}\frac{{RA}_{signal}}{1+{RA}_{signal}+f_{0}e^{-\lambda(x_{f}-x)}}, & 0<x<x_{f}-40 \\ k_{max}, & \text{elsewhere} \end{matrix} \right.$$

where ${RA}_{signal}={(\gamma[RA-R])}^{4}.$

The computational domain is a rectangle of dimension 500 μm x 50 μm. The parameters used in the simulation are directly from our previous studies and shown in Supplementary File 1B.

**Supplementary File 1B – Modeling parameters**

| Parameters | Values | Reference |
| --- | --- | --- |
| $\text{β}$ | 0.1 | White et al., 2007 |
| $\text{k}_{\text{p}}$ | 0.0001, 1000 | White et al., 2007 |
| $\text{k}_{\text{max}}$ | 1 | --- |
| $\gamma$ | 100 | --- |
| $\text{β}_{\text{0}}$ | 400 | White et al., 2007 |
| $\text{λ}$ | 0.1$\text{μm}^{\text{-1}}$ |  |
| n | 4 | --- |
| $\text{x}_{\text{f}}$ | 400$\text{μm}$ | White et al., 2007; Zhang et al., 2012 |
|  | 0.01 | --- |
|  | 100 | --- |
| V(x) | $\left\{ \begin{matrix} \text{10}^{\text{-5}}\text{μM}\text{sec}^{\text{-1}} & \text{if x>}\text{x}_{\text{f}}\text{-120} \\ \text{0} & \text{elsewhere} \end{matrix} \right.$ | --- |
| $r_{on}$ | 3$\text{μM}^{\text{-1}}\text{sec}^{\text{-1}}$ | Cai et al., 2012 |
| $r_{off}$ | 0.001$\text{sec}^{\text{-1}}$ | Cai et al., 2012 |
| $m_{on}$ | 3$\text{μM}^{\text{-1}}\text{sec}^{\text{-1}}$ | Cai et al., 2012 |
| $m_{off}$ | 0.0013$\text{sec}^{\text{-1}}$ | Cai et al., 2012 |
| $j_{\alpha}$ | 2$\text{μM}^{\text{-1}}\text{sec}^{\text{-1}}$ | Cai et al., 2012 |
| $j_{\beta}$ | 1$\text{μM}^{\text{-1}}\text{sec}^{\text{-1}}$ | Cai et al., 2012 |
| $bp_{deg2}$,${bp}_{deg1}$ | 0.0001$\text{sec}^{\text{-1}}$ | Cai et al., 2012 |
| $V_{R}$,$V_{BP}$ | ${10}^{-6}\text{ μM}\text{sec}^{\text{-1}}$,${10}^{-7}\text{/2 μM}\text{sec}^{\text{-1}}$ | Cai et al., 2012 |
| $r_{deg1}$,$r_{deg2}$ | 0.0001$\text{sec}^{\text{-1}}$ | --- |
| $\varepsilon_{in},\varepsilon_{out}$ | 0.006,0.08 |  |
| $\text{D}_{\text{RA}}$ | $\text{20}\text{μm}^{\text{2}}\text{/sec}$ | White et al., 2007; Zhang et al., 2012 |

Parameters are based on (White et al., 2007; Cai et al., 2012; Zhang et al., 2012).
